# First Complete Genome Sequence of Pepper mild mottle virus from Chili Pepper in the United States

**DOI:** 10.1128/genomeA.00331-18

**Published:** 2018-05-03

**Authors:** Kathryn Secrist, Akhtar Ali

**Affiliations:** a Department of Biological Science, The University of Tulsa, Tulsa, Oklahoma, USA

## Abstract

Pepper mild mottle virus (PMMoV) was first reported as a latent strain in the United States and then later reported in other countries around the world. Here, we report the first complete genome sequence of a PMMoV isolate (BL14) that was collected from chili pepper during the 2014 growing season in Oklahoma.

## GENOME ANNOUNCEMENT

Chili and bell peppers are economically important cash crops grown in the United States (1). Viral infection causes destructive effects in Capsicum species, resulting in significant economic loss (2). Pepper mild mottle virus (PMMoV) is one of the important pepper viruses that infect pepper plants worldwide. PMMoV (genus Tobamovirus, family Virgaviridae) (3, 4) is a single-stranded positive-sense RNA virus with a 6.3-kb genome and is one of the viruses highly transmitted by seeds (5).

PMMoV was first identified in the early 1950s as a latent strain in the United States (5) and then in 1984 as a distinct strain in Italy (6). Since then, it has been reported in many countries around the world. In the United States, PMMoV has been isolated from peppers in several states, including Colorado, Florida, Georgia, Oregon (7, 8), South Carolina (6, 7, 9, 10), and Texas (11). Recently, we reported for the first time a PMMoV infection during the 2014 growing season in Oklahoma (12).

Although PMMoV is one of the important pepper viruses, to our knowledge no complete genome sequence has been reported from the United States. In this study, we report the first complete genome sequence of a PMMoV isolate collected from a commercial pepper field in Oklahoma.

In our previous work, a sample showing typical PMMoV symptoms was collected in Blaine County, Oklahoma (designated PMMoV isolate BL14), and brought to the laboratory. Virus-infected leaves were tested by DIBA against the antisera of PMMoV and mechanically inoculated on the leaves of healthy pepper seedlings using extracted sap from infected tissue (12). The virus culture was maintained in peppers in the growth chambers.

Total RNA was extracted according to the Tri-Reagent procedure (13) from young leaves of pepper plants systemically infected with PMMoV isolate BL14. Six pairs of overlapping primers were designed from the complete genome sequences of PMMoV isolates available from GenBank (https://www.ncbi.nlm.nih.gov). Seven genome fragments were amplified by reverse transcription-PCR, as described previously (13). PCR products of each fragment were confirmed on 1% agarose gel and then purified from the gel and cloned into a pGEM-T Easy vector. At least three to five recombinant clones for each PCR fragment were sequenced in both forward and reverse directions using an Applied Biosystems model 3130 analyzer at the Department of Biological Science, The University of Tulsa, Oklahoma.

Sequence alignment to generate consensus nucleotide sequences of each fragment was done using both Clustal W (14) and Muscle alignment (15). The complete genome of PMMoV was generated by joining overlapping sequences among adjacent fragments using both Clustal W and Muscle alignment. Sequence alignments were generated using MEGA version 7.0 software (16).

The complete genome sequence of PMMoV isolate BL14 is 6,353 nucleotides long. Nucleotide BLAST searches showed that isolate BL14 shared 94% to 99% nucleotide sequence identity with available PMMoV complete genomes in the GenBank database. Maximum nucleotide (99%) and amino acid sequence similarities were observed with a Spanish-S isolate (GenBank accession no. M81413).

### Accession number(s).

The complete genome sequence of PMMoV isolate BL14 was deposited in GenBank under the accession no. MH063882.
